# The management of difficult intubation in infants: a retrospective review of anesthesia record database

**DOI:** 10.1186/s40981-015-0020-7

**Published:** 2015-10-16

**Authors:** Junko Aida, Yutaka Oda, Yoshihiro Kasagi, Mami Ueda, Kazuo Nakada, Ryu Okutani

**Affiliations:** Department of Anesthesiology, Osaka City General Hospital, 2-13-22 Miyakojima-hondori, Miyakojima-ku, Osaka 534-0021 Japan

**Keywords:** Infants, General anesthesia, Airway, Tracheal intubation, Difficult intubation, Cormack-Lehane grade

## Abstract

We retrospectively reviewed the anesthesia records of infants < 1 year of age for elucidating the incidence of difficult intubation and airway management in a single general hospital. The electronic data records from a total of 753 consecutive anesthesiological procedures in 513 different infants were analyzed. After excluding data with a lack of records of laryngoscopic findings, a total of 497 procedures (389 different infants) with either remarks of difficult intubation (requiring > 10 min for tracheal intubation) or records of Cormack-Lehane grade were included. Demographic data are median age 5 (range, 0–11) months, height 61 (33–84) cm, body weight 6.0 (1.1 − 11.8) kg. The number of cases with ASA physical status I, II, III and IV was 182 (36.6 %), 135 (27.3 %), 177 (35.5 %) and 3 (0.6 %), respectively. Cormack-Lehane grade 1, 2, 3 and 4 was seen in 450 (90.5 %), 32 (6.4 %), 6 (1.2 %) and 6 (1.2 %) cases, respectively.

Document of difficult intubation was found in 12 cases (2.4 %, 10 different infants) with a lack of record of Cormack-Lehane grade in 3 cases. Of these 10 infants, nine had multiple congenital anomalies including heart diseases and cleft palate. Without premedication, general anesthesia was induced with intravenous midazolam or sevoflurane in the 12 cases. Tracheal intubation was performed after disappearance of spontaneous respiration except three cases who were intubated in the awake state or under sedation. Elapsed time from induction of anesthesia to intubation was 17 (14–29) min. Although mask ventilation was adequate in all cases, two cases (one infant) developed hypoxia and bradycardia during tracheal intubation. No remarkable decrease of SpO_2_ or bradycardia less than 100 bpm was detected in other cases. In conclusion, we found difficult intubation in 2.4 % of infants undergoing general anesthesia. Although muscle relaxants are useful for facilitating tracheal intubation, it should be carefully used with the preparation of other airway devices in infants with predicted difficult intubation.

## Background

Young children frequently develop hypoxia during induction of general anesthesia [1], which may lead to hypoxic brain damage, cardiac arrest and death [2], suggesting that tracheal intubation is crucially important for preventing these adverse events. Although the incidence of difficult intubation in children is believed to be lower than in adults [3], there is a paucity of data regarding it [4]. Moreover only few reports describe the details of anesthesia induction and intubation methods including choice of anesthetics, airway devices and the use of muscle relaxants in children with difficult intubation in a large data base. As the incidence of difficult intubation and laryngoscopy as well as development of hypoxia during induction of anesthesia is higher in infants < one year of age than older children [1, 4–6], it is clinically important to collect data in this age group of children. For elucidating the incidence of difficult intubation and airway management in a general hospital, we retrospectively reviewed anesthesia records and the profiles of children with difficult intubation.

## Case presentation

Following approval from the Osaka City General Hospital Research Ethics Review Board (no. 1408070, Oct 20, 2014), medical records of patients < one year of age receiving surgery under general anesthesia from May 2012 to May 2014 were retrospectively reviewed. Written informed consent was obtained from the parents of the 10 infants with difficult intubation. The review board has granted a waiver of informed consent from the guardians of the remaining infants with respect to the retrospective character of the study. Patients’ characteristics and details of anesthesia were collected from anesthesia records; information regarding inpatient status was collected from the hospital medical information system. General anesthesia was performed under standard monitoring by a staff anesthesiologist only or an anesthesiology resident supervised by a Japanese Society of Anesthesiologists Board Certified staff anesthesiologist. Methods of induction and selection of anesthetics were dependent on each attending anesthesiologist’s decision. Direct laryngoscopy was performed with a standard Macintosh blade sized appropriately. Other devices as Miller blade were not used. For difficult airway management, endotracheal intubation using a video laryngoscope or a flexible fiberoptic bronchoscope was a standard approach. Difficult intubation was defined as that tracheal intubation required more than 10 min [7]. In that case staff anesthesiologists performed laryngoscopy and tracheal intubation.

Overall, the electronic data records from 753 consecutive anesthesiological procedures in 513 different infants were analyzed. The database included all types of anesthesia for various types of surgery, diagnostic interventions requiring anesthesia. Owing to incomplete data, 34 case records were excluded from the analysis. No laryngoscopic findings were documented in cases with general anesthesia using a supraglottic airway device (LMA ProSeal^®^, *n* = 24), in face mask ventilation (*n* = 2), in infants already endotracheally intubated (*n* = 174), with tracheostomy (*n* = 18). Tracheal intubation was performed with a video laryngoscope twice (counted as two cases) in one infant undergoing neurosurgery. She was accompanied with craniosynostosis with Arnold Chiari malformation with restricted cervical range of motion. Fiberoptic intubation was performed in two infants with predicted difficult intubation, after confirming Cormack-Lehane grade 4 by direct laryngoscopy.

A total of 497 procedures (389 different infants) with either remarks of difficult intubation or records of Cormack-Lehane (CML) grade were included. Demographic data are median age 5 (range, 0–11) months, height 61 (33–84) cm, body weight 6.0 (1.1 − 11.8) kg. The number of cases with ASA physical status I, II, III and IV was 182 (36.6 %), 135 (27.3 %), 177 (35.5 %) and 3 (0.6 %), respectively. CML grade 1, 2, 3 and 4 was seen in 450 (90.5 %), 32 (6.4 %), 6 (1.2 %) and 6 (1.2 %) cases, respectively. Document of difficult intubation was found in 12 cases (10 different infants, Table 1) with a lack of record of CML grade in 3 cases. All of these infants except one with a tongue base cyst (case no. 7) had multiple congenital anomalies including heart diseases and cleft palate; ASA physical status was III or IV in eight cases. Of the 12 cases, five received cardiac surgery. An infant underwent surgery three times for congenital hydrocephalus and tetralogy of Fallot (nos. 1–3).

**Table 1:** Patients’ characteristics

No.	Age	Sex	BW (kg)	ASA PS	Diagnosis and complications	Surgery	Condition at intubation	Airway device	Cormack-Lehane grade	Agents used for induction and intubation
1	8 d	F	1.8	III	hydrocephalus, TOF, cleft lip, palate	Ommaya reservoir placement	anesthesia	laryngoscope	4	midazolam, rocuronium
2	2 m	F	2.7	III		VP shunt	anesthesia	laryngoscope	4	sevoflurane, fentanyl, midazolam, rocuronium
3	4 m	F	3.5	III		cardiac surgery	sedation	bronchoscope	−	midazolam, atropine, lidocaine
4	9 d	M	3.5	II	vein of Galen aneurysmal dilatation	embolism	anesthesia	laryngoscope	3	thiopental, fentanyl, rocuronium
5	20 d	M	1.7	IV	VSD, 18 trisomy	cardiac surgery	awake	laryngoscope	3	atropine
6	1 m	M	2.3	III	PDA	ligation	anesthesia	laryngoscope	4	sevoflurane, N_2_O, fentanyl, rocuronium
7	1 m	M	3.5	II	tongue base cyst	resection of cyst	anesthesia	laryngoscope	−	sevoflurane, N_2_O, pentazocine
8	2 m	F	1.8	III	HPS, 18 trisomy cleft lip, palate	Ramstedt’s operation	anesthesia	laryngoscope	4	thiopental, sevoflurane, rocuronium
9	2 m	M	2.6	III	DORV, cleft lip, palate	cardiac surgery	anesthesia	laryngoscope	4	sevoflurane, fentanyl, midazolam, rocuronium
10	2 m	M	3.4	III	TOF, cleft palate	cardiac surgery	sedation	bronchoscope	4	midazolam, lidocaine
11	2 m	F	5.6	II	craniosynostosis	cranioplasty	anesthesia	video laryngoscope	−	sevoflurane, fentanyl, rocuronium
12	10 m	M	6.8	II	Crouzon syndrome	tracheostomy	anesthesia	laryngoscope	3	sevoflurane, N_2_O, pentazocine, rocuronium

*ASA PS* American Society of Anesthesiologists physical status, *d* days, *DORV* Double outlet right ventricle, *HPS* hypertrophic pyloric stenosis, *m* months, *N*_*2*_ *O* nitrous oxide, PDA patent ductus arteriosus, *TOF* tetralogy of Fallot, *VP shunt* ventriculoperitoneal shunting, *VSD* ventricular septal defect

Cases nos. 1, 2 and 3 are the same patient. –: no records or no laryngoscopy

Premedication was not administered in these 12 cases. General anesthesia was induced with intravenous midazolam only or midazolam with fentanyl in cases with intravenous lines at the induction of general anesthesia, or with sevoflurane in cases without intravenous lines, followed by administration of rocuronium (8 cases) or pentazocine (1 case) before tracheal intubation using a laryngoscope after confirming adequate mask ventilation. Other intubation devices were not used in these cases. A video laryngoscope (AWS-S100L, Pentax^®^, Tokyo, Japan) with an introducer blade (ITL-N, MIC Medical Corp.^®^, Tokyo, Japan) was used instead of laryngoscope in a case of craniosynostosis accompanied with restricted cervical range of motion and spinal cord compression.

Tracheal intubation was performed in the awake state or under sedation in the remaining three cases. In a case of 20 days of age with 18 trisomy (no. 5), a 2.5-mm tracheal tube placed immediately after birth was exchanged for a 3.0-mm tube without anesthetics or sedatives because of air leakage during positive pressure ventilation. In other two cases tracheal intubation was performed using a bronchoscope under sedation with midazolam with spontaneous respiration, based on previous anesthesia records (no. 3) or findings of direct laryngoscopy (no. 10) showing CML grade 4.

In these 12 cases, elapsed time from induction of anesthesia to intubation was 17 (14–29) min. Although mask ventilation was adequate in all cases, one infant developed hypoxia and bradycardia during tracheal intubation at eight days and two months of age (nos. 1 and 2). She was successfully intubated using a bronchoscope under sedation with spontaneous respiration during anesthesia performed at 4 months of age (no. 3), with no resultant brain damage. She received anesthesia additional four times after one year of age. CML grade decreased from 4 at 4 months to 3 after 1 year of age and tracheal intubation was performed without difficulty using a stylet or a video laryngoscope. No remarkable decrease of SpO_2_ or bradycardia less than 100 bpm was detected in other cases.

### Discussion

In this retrospective data review, difficult intubation was found in 12 cases among the total number of 497 (2.4 %), which was more than two-fold higher than that reported previously in infants [4], although the details of patients such as complications and types of surgery in that report is not clear. Another report showed that the incidence of difficult laryngoscopy (Cormack-Lehane grade 3 or 4) in patients < one year (4.7 %) was significantly higher than in the older patients (0.7 %) [5]. The incidence of difficult laryngoscopy was significantly higher in children receiving cardiac surgery and oromaxillofacial surgery [5], although the number of patients undergoing such surgical procedures is not known.

Large number of infants with low body weight and multiple deformities classified as ASA physical status III, which is accompanied with high incidence of difficult laryngoscopy (CML grade 3 and 4) [5], included in the present study would be responsible for this relatively high incidence of difficult intubation. CML grade 3 or 4 was found in 12 cases (2.4 %, 6 cases each), comparable to the rate reported previously in the same age group [5]. However CML grade 3 does not necessarily correspond to difficult intubation [8]. Tracheal intubation was performed without difficulty in three cases with CML grade 3 among the 497 cases by experienced anesthesiologists using a stylet.

Besides younger than one year of age, several causes have been suggested as predictors of difficult laryngoscopy [3, 5, 6]. In the present report eight cases were accompanied with craniofacial deformities including cleft palate and craniosynostosis, five cases required cardiac surgery. Both of them are reported as predictors of difficult laryngoscopy [5]. Although Mallampati score is a reliable predictor of difficult laryngoscopy in children as well as in adults [5], precise evaluation would be difficult in infants.

Despite the variety of available agents, choice of methods and agents for induction of general anesthesia and tracheal intubation in pediatric patients is less established [9]. In the 12 cases with difficult intubation, midazolam only or midazolam with fentanyl were predominantly used as intravenous agents instead of propofol, which is most commonly used for anesthesia induction in patients < 18 years [5]. We avoided the use of propofol for preventing injection pain and cardiovascular depression. Fentanyl was also used in patients after induction of anesthesia with sevoflurane for suppressing sympathetic responses to tracheal intubation.

Most of the cases received rocuronium for tracheal intubation, suggesting that spontaneous respiration does not recover even in case of failed intubation. As muscle relaxants are effective for increasing the success rates of tracheal intubation and for preventing preoperative respiratory adverse events [10, 11], we routinely use it as reported elsewhere [12]; however, apnea easily develops hypoxia during tracheal intubation, as shown in our cases. Muscle relaxants should be carefully used with preparation of other airway devices. A video laryngoscope with a blade for neonates or infants, and a bronchoscope would be useful if tracheal intubation is difficult with a conventional laryngoscope. However fiberoptic intubation would be more difficult in infants than in adults because of smaller oral cavity, thin tracheal tube, and rapid decrease of oxygen saturation, which require profound skill.

There is repetitive inclusion of many infants who received general anesthesia repeatedly at different age; however, it will not influence the basic results of the present study. Children may require multiple surgical procedures for congenital anomalies with age-specific anatomy. Interestingly CML grade was 1 in more than 90 % of all cases, which was not changed during repeated anesthesia provided by different anesthesiologists. On the other hand CML grade was decreased from 4 to 3 in one, and form 3 to 2 in two infants during anesthesia performed later, suggesting that the degree of difficult intubation changes with the development of age.

There are several limitations in this report. First this is a retrospective review of anesthesia records in a single hospital with limited number of cases. Second, the assessment of CML findings is based on the subjective view of the attending anesthesiologists, and we did not perform anatomical measurements for predicting difficult intubation such as thyromental distance.

## Conclusions

We found difficult intubation in 2.4 % of infants < one year of age undergoing general anesthesia. Most of the infants with difficult intubation were accompanied with congenital heart disease and/or cleft palate. Although muscle relaxants are useful for facilitating tracheal intubation, it should be carefully used with the preparation of other airway devices in infants with predicted difficult intubation.

## Consent

Written informed consent was obtained from the patients for publication of this case report. A copy of the written consent is available for review by the Editor-in-Chief of this journal.
